# Field evaluation of low-dose warfarin baits to control wild pigs (*Sus scrofa*) in North Texas

**DOI:** 10.1371/journal.pone.0206070

**Published:** 2018-11-07

**Authors:** Richard M. Poché, David Poché, Greg Franckowiak, Daniel J. Somers, Lindsay N. Briley, Batchimeg Tseveenjav, Larisa Polyakova

**Affiliations:** Genesis Laboratories, Inc., Wellington, Colorado, United States of America; Centre for Cellular and Molecular Biology, INDIA

## Abstract

Wild pigs (*Sus scrofa*) are a highly detrimental invasive species that occupy a rapidly expanding range within the United States. In Australia, field trials evaluating baits containing 0.09% warfarin resulted in wild pig population reduction >95%. The objective of this study was to conduct an EPA-approved field trial to evaluate the use of bait containing low-dose warfarin (0.01% and 0.005%) in reducing wild pig numbers in Texas. An 8-week field test was conducted in the panhandle ~100 km southeast of Amarillo. Two ~8 km^2^ treatment plots were selected and each presented with either 0.01% or 0.005% warfarin baits. One control plot (~8 km^2^) was presented placebo. The baits were delivered using 30 species-specific feeders per plot (*n* = 90) that kept wildlife from accessing the toxicant. Pig movements and feed consumption were monitored during pre-treatment, treatment, and post-treatment periods. All pigs with VHF transmitters within the 0.005% warfarin-treated plot (*n* = 14) succumbed to the warfarin (100% mortality). Overall, 35 wild pigs were found dead from warfarin toxicosis, within both treatment plots. Total feed consumption by wild pigs was reduced by ~97.8% and ~96.2% for the 0.005% and 0.01% warfarin baited plots, respectively, indicating the absence of pigs was a result of toxic bait consumption. Results of 97 systematic searches of the treatment plots indicated no warfarin-induced non-target wildlife fatalities. Warfarin residues in wild pig livers averaged 3.69 mg/kg (*n* = 13) and 2.89 mg/kg (*n* = 9) for pigs recovered within the 0.005% plot and 0.01% warfarin plot, respectively. This study is the first efficacy field evaluation of a wild pig toxicant conducted in the US. The results suggest low-dose warfarin bait, presented in species-specific feeders, can effectively reduce wild pig numbers and pose minimal risk to non-target wildlife and domestic animals. A product containing warfarin may provide another management tool in reducing wild pig problems.

## Introduction

The wild pig (*Sus scrofa*,) is an invasive species, with one of the widest global distributions of any animal [1], extending to all continents except Antarctica [2,3]. In many parts of the world, wild pig population expansion results from human introduction [2]. Damage associated with wild pigs has been reported for centuries [3] and the Species Survival Commission of the World Conservation Union has listed them as being among the 100 worst invasive alien species globally [2].

Wild pigs are habitat and dietary generalists and as a result may compete with native wildlife [4] such as collared peccaries (*Pecari tajacu*) and white-lipped peccaries (*Tayassu pecari*) in North and South America [5,6]. Wild pigs also cause extensive economic damage to agriculture and livestock industries [7–9], and prey on wildlife and domestic animals, including endangered species [10,11]. Additionally, wild pigs carry numerous diseases, with meat processed in commercial plants in Texas having been found positive for multiple disease pathogens, including influenza A, *Leptospira*, *Trichinella*, *Toxoplasma*, and *Brucella* spp. [12,13]. These authors indicated 48.9% of tested pigs were positive for antibodies of one or more serovars of *Leptospira* spp. and 9.0% for antibodies of *Toxoplasma gondii*.

In the North America, wild pig abundance has increased significantly since 1990, and this increase is largely a result of an accelerated birth rate, habitat and foraging adaptability, and continued introduction for sport hunting [14–16]. Wild pigs are now confirmed in 44 US states [17] and in parts of Canada, such as Saskatchewan, which were previously believed to be uninhabitable [18]. This distribution will likely continue to increase if population control cannot be achieved [19]. Control techniques used for reducing wild pig populations have included fencing and diversions, trapping, hunting, aerial gunning, and immunocontraception [20–26]. Many of these methods are considered costly and labor-intensive [8, 20]. Hunting can locally reduce wild pig numbers and provide economic benefits but cannot effectively reduce populations on a large scale [20, 27, 28]. Previous estimates suggest the annual rate of wild pig increase to be ~86% [29] during which it is estimated that on average only ~23% of pigs are harvested through sport hunting [30].

Toxic bait is considered a potential option for reducing wild pig abundance [31]. The advantages of baits are the relative low cost and potential for large-scale application. The method is less labor intensive and can be efficacious [32]. Compound 1080 (Sodium fluoroacetate) [33], warfarin [34, 35], and sodium nitrite [36, 37] were evaluated for wild pig control in Australia and showed promise. While compound 1080 is approved for use in Australia, it has been banned for use in rodent control in the U.S. since 1989 and use of the compound in pest control is restricted to toxic collars for controlling coyotes (*Canis latrans*) [38].

Warfarin is a first-generation anticoagulant which, at low doses, is used medicinally in humans to treat thrombosis [39]. Warfarin was approved by the Food and Drug Administration (FDA) for medicinal use since 1954 [40] and by the EPA for use as a rodenticide since 1952 [41]. When used as a rodenticide, a latency period occurs prior to symptom onset [42–44], which decreases the likelihood of bait shyness, encouraging continued consumption [28]. The average days until death of laboratory-bred house mice *(Mus musculus*) and rats (*Rattus norvegicus*) exposed to warfarin bait (0.025%) are 5 and 6 days, respectively, which are similar to that of far more toxic second-generation anticoagulants [45]. Warfarin is metabolized quickly, reducing the risk of secondary exposure to scavengers [22] and has an antidote, Vitamin K1 [39,46].

Research has shown warfarin to be effective as a wild pig toxicant, with an acute oral LD_50_ of 3 mg/kg body weight, compared to 50–100 mg/kg for adult rats (*Rattus rattus*, *Rattus norvegicus*) [43]. Results of a pen study, conducted in Kingsville, Texas demonstrated 100% mortality of wild pigs within 8 days when exposed to a bait containing warfarin (0.005%) for 5 consecutive days [47]. In two field trials in Australia, wheat treated with 0.13% and 0.09% warfarin was estimated to reduce wild pig abundance in treated areas by ~94% and ~99%, respectively [34,48]. These concentrations were ~5 and ~3.5x the EPA-recommended concentration of commercial rodent baits in the US [41,45]. Given the sensitivity of wild pigs to warfarin, it is reasonable to assume, not only that the warfarin concentrations utilized in the Australian trials were unnecessarily more toxic than required, but that wild pig populations in the U.S may be sensitive to warfarin at concentrations as low as 0.005%.

A field study was conducted in north Texas, the primary objectives being to 1) evaluate the efficacy of paraffin baits (0.005% and 0.01% warfarin), applied in feeders, and 2) assess the primary and secondary toxicity risks of warfarin baits to non-target wildlife. Authorization to begin field testing of the warfarin baits was obtained on June 24^th^, 2014 from the EPA in the form of an Experimental Use Permit (72500-EUP-2). Subsequently, the Texas Department of Agriculture approved of the field experiment on October 7^th^, 2014. While previous researchers have evaluated the use of warfarin and sodium nitrite baits under pen conditions [37, 47], to our knowledge, this is the first field study to evaluate the use of a wild pig toxicant (warfarin) in controlling wild pig numbers in the United States. The results of this study should provide insights into how warfarin baits could work under field conditions in the Texas Panhandle.

## Materials and methods

All activities involving animals for this study were reviewed and approved by the Institutional Animal Care and Use Committee (IACUC) at Genesis Laboratories and followed the Animal Welfare Act and Genesis Laboratories (Wellington, CO) Institutional Animal Care and Use policies (protocol: 15002).

The EPA granted an Experimental Use Permit (72500-EUP-2) to allow for field testing. The Texas Department of Agriculture approved the field experiment on October 7, 2014.

### Study area

**Fig 1:**
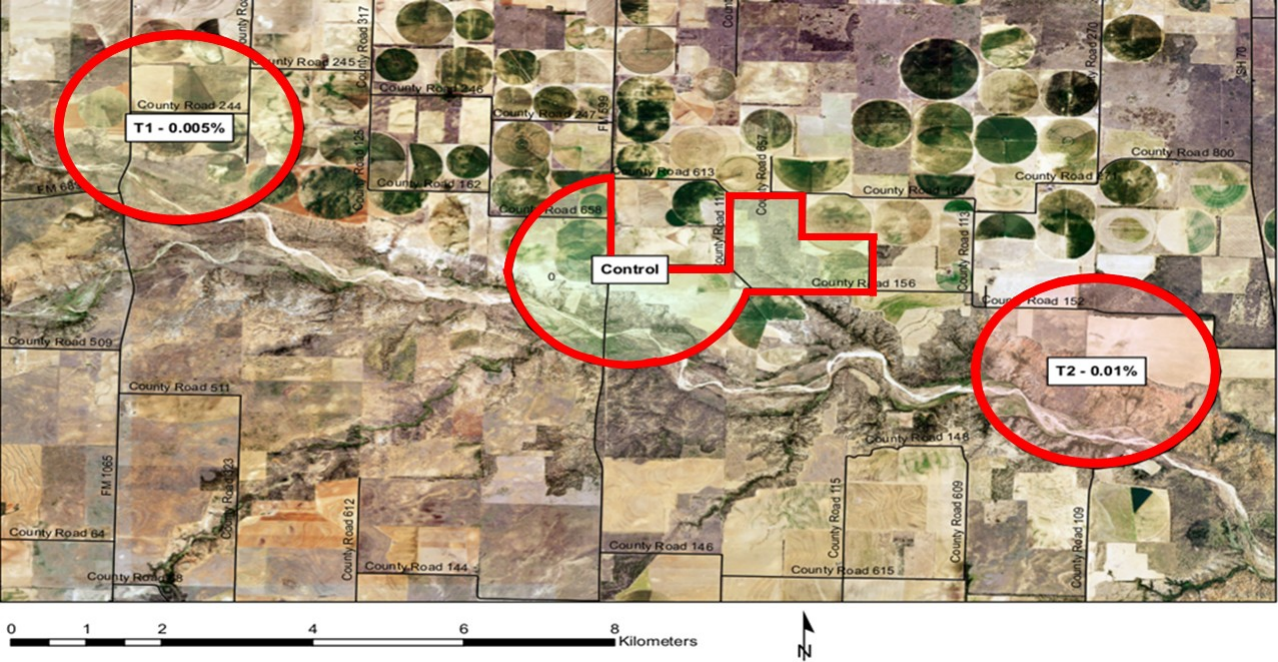
Map of three study plots, located approximately 50 km east of Plainview, Texas, used during pilot field study conducted from April 9^th^-June 9^th^, 2015. The map was generated in ArcGIS using ArcMap with a publicly-available National Agricultural Imagery Program base layer (Source: https://gdg.sc.egov.usda.gov/GDGHome_DirectDownLoad.aspx. Accessed: 7/23/2015).

An 8-week field study was conducted from April 9^th^—June 9^th^, 2015 in north Texas, ~100 km southeast of Amarillo. Three study plots were selected based on wild pig activity as indicated by sightings, tracks, wallows, crop or pasture damage, and trails. Each plot was ~8 km^2^ and on private land comprising agricultural fields, pastureland, and scrub and trees with sufficient escape cover for wild pigs. Primary agricultural crops in the region include cotton, sorghum, and peanuts, all of which are commonly damaged by wild pigs [7]. Two treatment plots were discerned and presented with 0.005% or 0.01% warfarin bait. A third plot of equal size served as the control in which placebo was applied (Fig 1). Permission was received from the landowners prior to plot selection and study initiation. The plots were individual private properties which were completely independent of one another, and whose boundaries were separated by several kilometers.

### Wild pig bait

Paraffin bait formulations were manufactured at Scimetrics Limited Corp (Wellington, CO). The bait matrix was developed based on the result of a previous wild pig pen study [47] that indicated its acceptability. Paraffin baits were selected since these are more weather-resistant and ensure stability of the warfarin [49]. A fat-soluble Keystone Blue dye (Keystone Innovative Color Solutions, Chicago, IL), which stains internal and subcutaneous fat, was incorporated into the bait to confirm bait consumption by wild pigs. The dye has previously been used to mark various rodents (*Rattus norvegicus*, *Spermophilus beecheyi*, *Thomomys bottae*) and was noted to not adversely reduce bait acceptance by the target species [50]. The warfarin concentration in the two baits were 0.005% and 0.01% warfarin and the baits were manufactured using a commercial extruder which cut bait slices into approximately 14-g parcels. A warfarin-free paraffin bait was manufactured as a placebo bait.

### Study periods

The study was divided into three periods, preceded by a feeder-conditioning time of ~3 weeks:

#### Feeder conditioning

Feeders were secured with the doors open and commercial corn was placed inside to entice pigs to feeders. Pigs were allowed free access for three weeks before the study initiated.

#### Pre-treatment

Placebo was presented within feeders on all plots for 3 weeks (April 9^th^-April 30^th^, 2015) to establish baseline feed consumption. Baseline feed consumption is defined as the average weekly consumption of placebo from feeders per individual plot occurring during the pre-treatment period.

#### Treatment

Baits containing warfarin replaced placebo in feeders within both treatment plots for about 4 weeks (May 1^st^ -May 28^th^, 2015). Feeders within the control plot continued to be provided placebo.

#### Post-treatment

Warfarin bait in feeders within both treatment plots was replaced with placebo bait for ~2 weeks (May 29^th^ -June 9^th^, 2015). Feed consumption was monitored after the baiting period which, when compared with baseline feed consumption, would provide an indication of efficacy against wild pigs.

### Feeders and bait presentation

**Fig 2:**
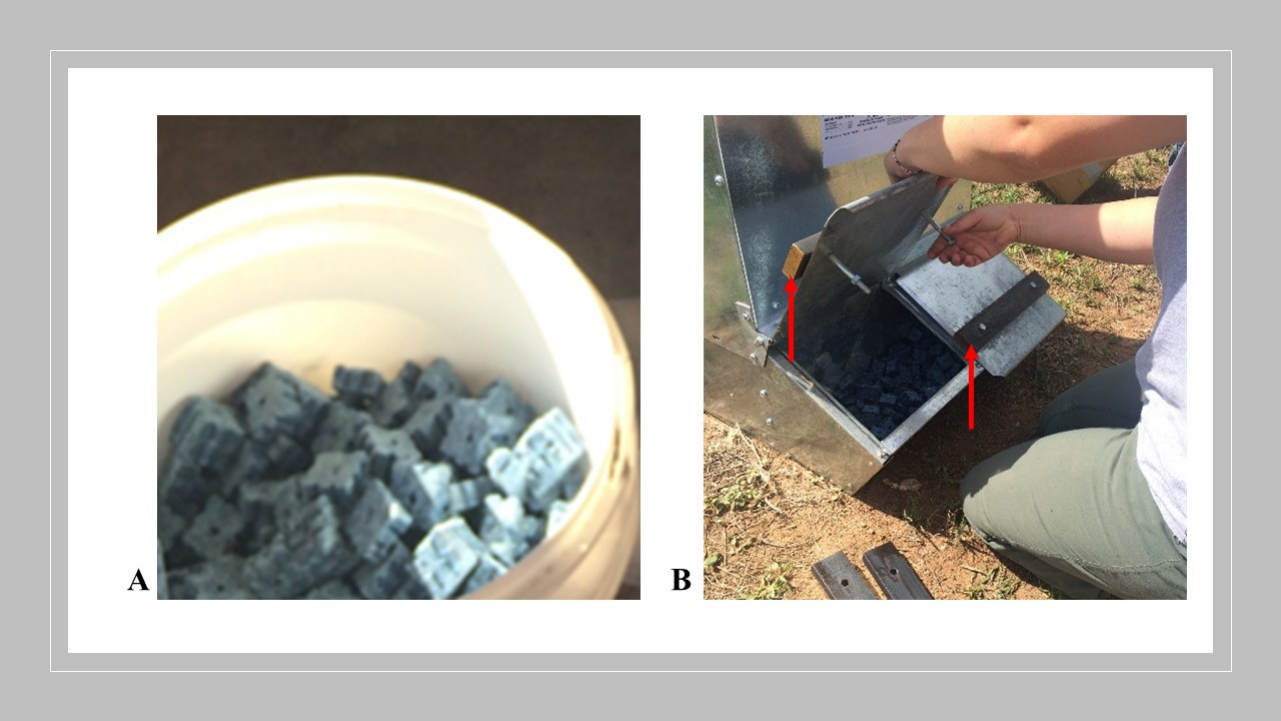
(A) Paraffin extruded wax blocks containing 0.005% warfarin; and (B) commercial pig feeder with custom-built ~4.5 kg weights attached to doors (red arrows) to limit non-target access.

Bait and placebo were presented in 90 commercially available double-door feeders (Brower Equipment #22H feeders, Houghton, IA; Miller Manufacturing #HGFD feeders, Glencoe, MN). The dimensions of each feeder were approximately 61.6 cm L x 50.2 W x 90.9 H. Feeder placement within the plots was based on wild pig activity as noted by tracks, wallows, or nearby pig damage to crops. Ten (10) clusters, of three feeders each, were placed within each of the three plots. Twenty-two (22) kg of feed (commercial corn, placebo, bait) was presented in each feeder at the start of each study period. Feed was replenished *ad libitum* with 22.0 kg being added to each individual feeder each time. At the end of each study period (Pre-treatment, treatment, post-treatment) all feed within each individual feeder was removed and weighed to the nearest 0.1 g. Clusters were placed in areas with evidence of recent pig activity (i.e. rooting damage). The cluster approach was used to ensure pigs in larger sounders would access feed or bait before moving away from feeders, effectively reducing competition for available feed or bait. Thus, the relative distance between clusters was not uniform. No feeders were <100 m from one another. Feeders were secured using bailing wire, either to trees or T-posts driven into the ground. During the treatment period, custom-built ~4.5 kg metal weights (IMS, Plainview, TX) were bolted to the feeder doors with the intent to exclude non-target wildlife (Fig 2). The design of the feeders protected the contents from rain and other falling debris.

For safety, and in accordance the Experimental Use Permit requirements, feeders were positioned in areas away from fields and pastures with livestock to ensure livestock would not access the bait. When possible, feeders were placed in shade to protect against heat and hidden from obvious view by humans, to avoid possible vandalism. GPS coordinates were taken at each feeder placement to use in data analysis and pig activity evaluation. Feeders were checked weekly and refilled *ad libitum*, and the amount presented was recorded (0.1 g) during each presentation. At the end of each study period, remaining bait or placebo in each feeder was collected and weighed. Feeders on the treatment plots were checked for bait spillage from wild pig feeding activity during the treatment period. Spilled bait was collected and weighed.

### Trapping and radio telemetry

Wild pigs were trapped and equipped with radio tracking devices to monitor movements and mortality. Trapping took place over 39 consecutive days (March 22^nd^ -April 30^th^, 2015) using 10 pig box-traps (2.4m x 1.2m x 0.9m; Voorhies Outdoor Products, LLC). Traps were baited with fermented corn, rather than the bait formulation, and were placed away from feeders (>100 m), which likely reduced any effect on pre-exposure or bias towards bait consumption. Each trapped pig in good health weighing ~<55 kg had a single very high frequency (VHF) transmitter (M3620 Mammal Ear Tag; Advanced Telemetry Systems, Isanti, MN) attached at the base of one ear. Each pig weighing ~>55 kg had a global positioning system (GPS) satellite-tracking collar (LoTek Wireless, Inc. Newfoundland, Ontario Canada, or North Star Science and Technology, LLC. King George, VA) secured safely around the neck. For the safety of animals and personnel, wild pigs were chemically immobilized with an intramuscular dosage of a mixture of Telazol and Xylazine (1.0 cc/18 kg) [51] before attaching transmitters. The study plots were considered sufficient if >10 wild pigs were trapped and equipped with transmitters within each plot.

Radio-tagged pigs were monitored daily, using VHF receivers (R2000 and R4000 receivers, Advanced Telemetry Systems, Isanti, MN) and handheld Yagi antennas, from the day that the tag was applied until found dead or until study termination (end of post-treatment). Both VHF ear tags and GPS collars were equipped with mortality sensors. When a mortality signal was detected, the transmitter was tracked to the location. At that time, it was determined whether the ear tag had detached from the pig or a carcass was present. When a carcass was recovered, it was necropsied and examined for signs of bait consumption by noting blue coloration in the internal fat or gastrointestinal track and hemorrhaging of internal organs. Surviving animals were not necropsied because of difficulties in retrieving these free-ranging animals in an open system.

### Trail cameras

Motion-sensing trail cameras (Primos Hunting Truth Cam 40; Primos Hunting, Flora, MS), were positioned at each feeder cluster, either strapped to a tree or T-posts at a height of ~1 m and positioned ~5 m from the feeders, to record visitations by pigs and non-target wildlife. The cameras were capable of capturing daylight images as well as nighttime infrared photos with the date and time. Images were examined for presence of wild pigs and non-target wildlife at feeder clusters. Using trail camera images, wild pig activity at clusters was determined within all three test plots to confirm that pigs were accessing the feeders and consuming the bait or placebo. A randomly assigned 3-day census period during pre-treatment was used to further confirm the presence of the target species [52].

Non-target wildlife visiting clusters were examined daily at feeder locations during the pre-treatment and treatment periods using trail camera images. Visitations were defined as the entire duration of appearance of an individual or group of animals on camera. For each visitation, the date and time of first appearance, species, and number of individuals, and observed behavior (approaching or opening feeder doors) were recorded. For analyses, all species of bird, small rodent, other small mammal, and large mammal (except of deer species and raccoons) species were categorized into the following groups: bird species, small rodent species, small mammal species, and large mammal species. Non-target animals were not marked and therefore we were unable to identify unique individuals.

#### Carcass search for target and non-target species

Carcass searches were conducted on all plots using a systematic grid design. To look for carcasses, the entire 8 km^2^ area of each plot was searched to ensure that any pigs or non-target wildlife fatalities were accounted for. Searchers were separated by ~10 m and walked in parallel lines across the area to detect target and non-target species carcasses. Animals equipped with radio transmitters were located and tissues collected for residue analysis. Treatment plots were searched daily for carcasses from the first day bait presentation was initiated until the end of the post-treatment period. All carcasses discovered on plots during searches were observed for signs of bait consumption. Necropsies were performed unless decomposition or scavenging activity were too advanced for observations to be made. Carcasses of non-target animals that were known to have been killed by gunshot from hunters (bullet wounds present) were excluded from the study. Liver tissue samples (~50 g) were collected from all carcasses unless a sample could not be recovered because of decomposition or scavenging activity. With pigs that consumed bait, liver tissue would be expected to contain the highest warfarin residues [53]. Samples were frozen and transported to Genesis Laboratories (Wellington, CO) for analysis of warfarin residue by High Performance Liquid Chromatography (HPLC). Five and seven carcasses from the 0.005% and 0.01% warfarin-baited plots, found to have consumed the warfarin bait, were monitored over time, using trail cameras positioned ~ 1 m high and ~10 m from the respective carcasses, to determine decomposition and scavenging rates. Images were examined for species identification of scavengers.

### Data analyses

We calculated the number of non-target wildlife feeder visitations per animal type per week during the pre-treatment and treatment periods. We did not estimate non-target activity during the post-treatment period as the objective was to ensure wildlife could not access the feeders when the toxicant was deployed. Differences in non-target wildlife visitation rates were compared between the pre-treatment and treatment periods using a non-parametric Wilcoxon Rank-Sum test (*p*<0.05). Additionally, we evaluated the dependence of the number of visitations on the study phase (pre-treatment, treatment) using a Pearson chi square test (*p*<0.05).

We estimated the efficacy of warfarin in reducing radio-tagged pig abundance (% mortality) and feed consumption. It is logical to assume that a significant reduction in radio-tagged pig abundance and feed consumption occurring at feeder clusters within the warfarin-baited plots would indicate a significant decline in the number of wild pigs within the larger population distributed within the treated areas. Any radio tagged pigs dying of non-warfarin-related causes were excluded from the analysis. We calculated the total feed consumption per plot during the pre-treatment and post-treatment periods (kg) (Total Presented–Total Remaining). We then estimated the average weekly consumption per plot for both the pre- and post-treatment periods (Total Consumed / No. Weeks). Efficacy of warfarin bait in reducing 1) radio tagged wild pig abundance and 2) feed consumption by wild pigs, was estimated by comparing values before bait application with those post-application using a basic efficacy formula presented below:

$$Efficacy=\left(\frac{n\;pre-n\;post}{n\;pre}\right)\times 100$$

Where: *n pre* = Number of Pre-treatment wild pigs

*n post* = Number of wild pigs remaining at test termination

Weekly placebo consumption at feeder clusters were compared between pre-treatment (baseline) and post-treatment periods to determine if warfarin bait resulted in a decrease in feed consumption, which would indicate a possible reduction wild pig abundance. A reduction in wild pig abundance or feed consumption exceeding 1) the 70% reduction required for EPA registration [54] and 2) the 86% annual rate of population increase reported by [29] would support use of the product for control of wild pigs in Texas. Weekly consumption was calculated to account for variation in study period duration. Data were log-transformed [log (n+1)] to reduce variation and normalize distribution of the data, allowing for use of parametric tests. Differences in bait consumption occurring during pre-treatment, treatment, and post-treatment periods were compared within the study plots using an Analysis of variance (ANOVA) followed by *post hoc* Tukeys W procedure. All above statistical analyses were performed using JMP Statistical Software: Version 13 (Cary, NC, U.S.A).

#### Good Laboratory Practice Standards

This study was conducted according to the EPA (1998) Good Laboratory Practice Standards (GLP) which are required for Federal Insecticide, Fungicide, and Rodenticide Act (FIFRA) pesticide registration (Title 40. Protection of Environment, Code of Federal Regulations Part 160 Good Laboratory Practice Standards). An independent Quality Assurance unit monitored and inspected all components of the study.

### Ethical guidelines for use of animals

All activities involving animals for this study were reviewed and approved by the Institutional Animal Care and Use Committee (IACUC) and followed the Animal Welfare Act and Genesis Laboratories (Wellington, CO) Institutional Animal Care and Use policies (protocol: 15002).

## Results

### Bait consumption

During the treatment period, a total of 418 kg and 356.6 kg of bait was provided to wild pigs on the 0.005% and 0.01% warfarin-bait plots, respectively. The application rate of warfarin (g/km^2^) was estimated to be ~2.6 g and ~4.5 g for the 0.005 and 0.01% warfarin plots respectively. Bait consumption during the treatment period totaled 150 kg (7.5 g warfarin) and 78.3 kg (7.8 g warfarin) within the 0.005 and 0.01% warfarin plots, respectively (Table 1**)**. During the treatment period, bait spillage outside feeders was collected with ~466.3 g (~23.3 mg warfarin) and 210.1 g (~21 mg warfarin) being collected within the 0.005 and 0.01% warfarin treatment plots, respectively.

**Table 1:** Summary of the amount of bait applied within two ~ 8 km^2^ plots (T1, T2) during the treatment period.

Study Plots (8 km^2^ each)	Applied			Consumed			Spillage	
	Bait (kg)	Warfarin (g)	Warfarin (g/km^2^)	Bait (kg)	Warfarin (g)	Warfarin (g/km^2^)	Bait (g)	Warfarin (g)
(0.005% Warfarin)	418	20.9	2.6	150	7.5	0.938	466.3	0.023
(0.01% Warfarin)	356.6	35.7	4.5	78.3	7.8	0.975	210.1	0.021

### Trapping and radio telemetry

Sixty-one (61) captured wild pigs were fitted with radio-tracking devices during the 39-day trapping period before study initiation, with VHF and GPS transmitters applied to 50 and 11 pigs, respectively **(**Table 2**)**.

**Table 2:** Number of trapped wild pigs within each plot fitted with radio tracking devices and number of pigs losing VHF tags or experiencing non-warfarin bait mortality.

	Study Plots			
	0.005% Warfarin	0.01% Warfarin	Control (untreated)	Total
VHF Tags Attached	17	19	14	50
GPS Collars Applied	4	7	0	11
Total Applied	21	26	14	61
Dropped VHF Tags	5	5	4	13
Non-bait Fatalities	2	7	4	13
Remaining pigs for Analysis	14	15	6	35

#### Target and non-target species visitation by trail camera images

**Fig 3:**
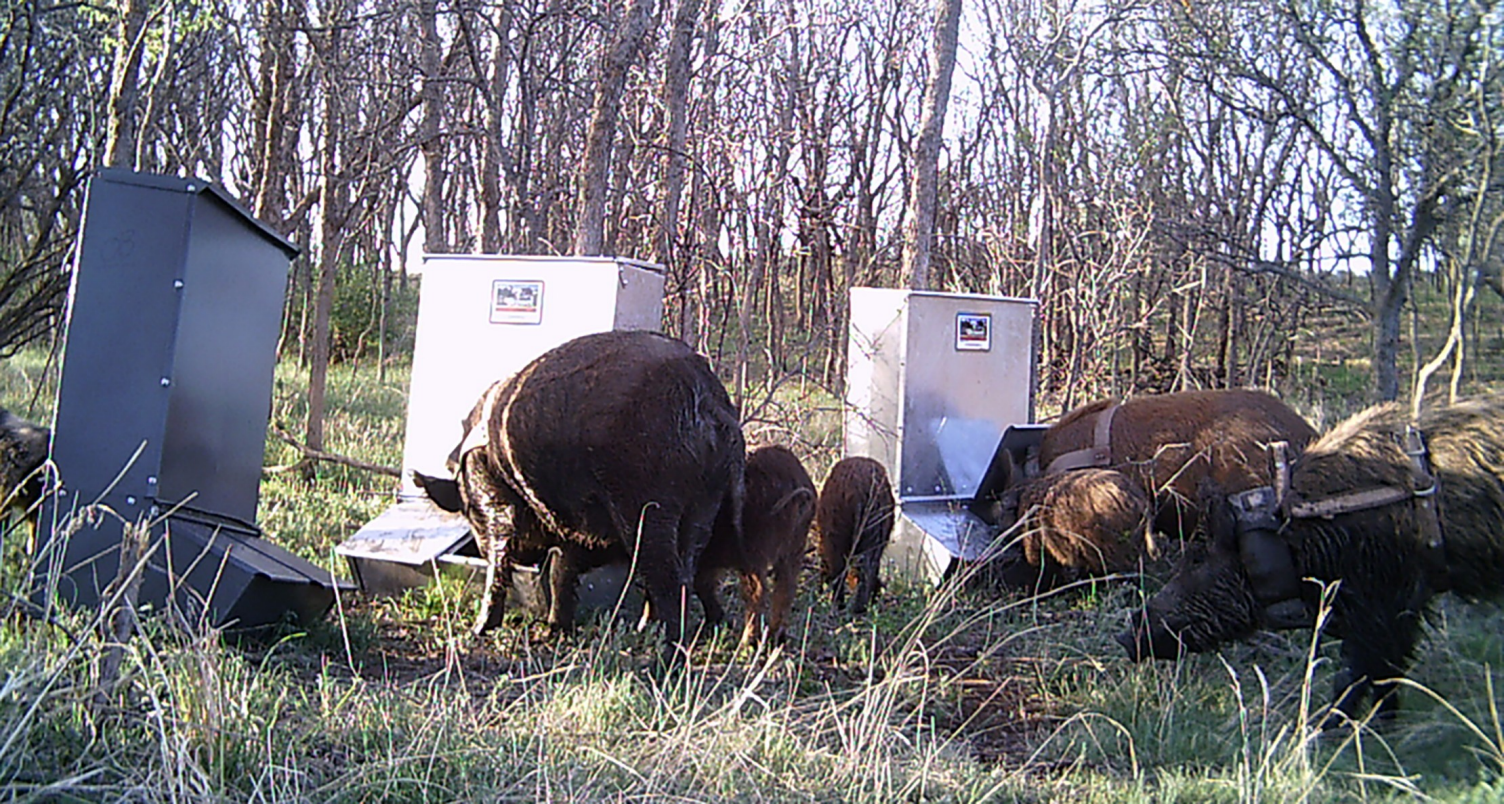
Wild pigs investigating and lifting doors to commercial feeders. GPS collars can be seen on the three large pigs.

In total, 344 pigs were observed on camera during the 3-day pre-treatment census period. Pigs lifted feeder doors easily to consume bait (Fig 3).

Numerous non-target species visited feeders, either passing through the area or approaching and investigating the feeders (Table 3). Multiple bird species were observed, including bobwhite quail (*Colinus virginianus*), Eurasian collared doves (*Streptopelia decaocto*), mourning doves (*Zenaida macroura*), turkeys (*Meleagris gallopavo*), Northern cardinals (*Cardinalis cardinalis*), Greater roadrunners (*Geococcyx californianus*), and several small migratory birds and passerines. Birds, as a group, accounted for 42 visitations (31%), or 14 visitations per week, within the control plot during pre-treatment. Small rodents (*Neotoma* spp.; *Peromyscus* spp.) were also observed near the feeder**s**. The raccoon (*Procyon lotor*) was the most commonly observed non-target species on the control plot, comprising 42 (14 per week) of the 139 total visitations (31%) recorded for non-target wildlife during the pre-treatment period.

**Table 3:** Summary of average weekly non-target wildlife species count by camera images at feeder clusters throughout the pre-treatment and treatment phases.

	Study Plots					
	0.005% Warfarin		0.01% Warfarin		Control	
Species Group	Pre	T	Pre	T	Pre	T
Raccoon (*Procyon lotor*)	8.7	0.3	24.3	8.5	14	3.3
Bird species	4.7	1	96	22.5	14	0.3
Small rodent species	12.3	0	56	14	7	0.3
Deer (*Odocoileus* spp.)	3.3	0	28.7	8	5.7	0
Small mammal species	2.0	0.8	5.0	3	5	0
Large mammal species	2.0	0.8	0.0	0.5	0.7	0
Snake (*Pituophis catenifer*)	0	0	1.0	0	0	0
Total	32.7	2.5	214.3	56	46.3	3.8

Non-target species were most prevalent within all plots during the pre-treatment period. A total of 98, 643, and 139 non-target wildlife visitations occurred within the 0.005% and 0.01% warfarin treated plots and the control, respectively. This amounted to an average of 32.7, 214.3, and 46.3 visitations per week. Although the treatment period was longer than the pre-treatment period, a significant decline in non-target wildlife visitation was observed within all plots, possibly a result of resident species losing interest in the feeders over time. A total of 10, 224, and 15 visitations occurred within the 0.005% and 0.01% treatment and control plots during the treatment period. This amounted to an average of 2.5, 56, and 3.8 visitations per week. Hence the weekly visitation rate was reduced by ~92, 74, and 92% during the treatment phase. The difference in weekly visitations between pre-treatment and post treatment were determined to be significant (Wilcoxon: *p* = 0.0052) with visitations being determined to be more frequent during pre-treatment (Pearson *X*^*2*^: *p*<0.0001).

#### Raccoon feeder access

**Fig 4:**
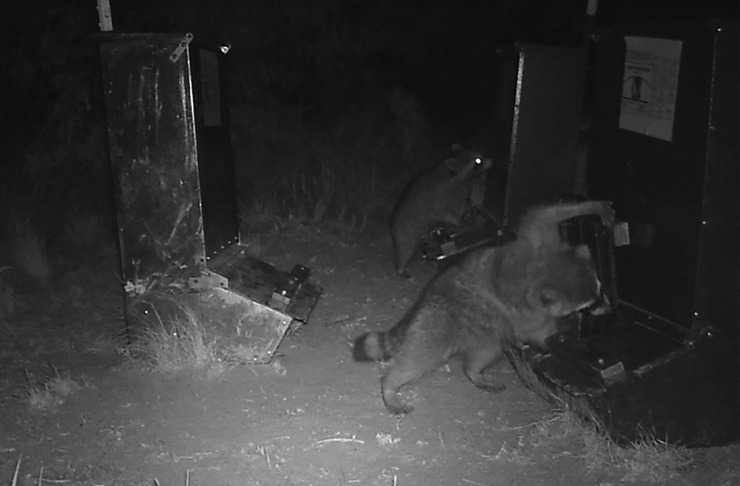
Raccoons opening commercial feeder doors with 4.5 kg weights attached.

Raccoons were the only non-target species to open feeder doors (Fig 4). Access attempts included lifting the feeder door with the snout or paws and attempting to open the top access lid. Of the 141 total visitations by raccoon to feeder stations on all plots during the pre-treatment period, raccoons opened feeder doors in 66 of 118 attempts. Feeder access by raccoons was far more common during pre-treatment than treatment and feeder entry was suggested to be dependent on study period (Pearson *X*^2^: *p* = 0.0073). Of the 99 visitations observed during the pre-treatment period within the 0.005% and 0.01% warfarin plots, raccoons opened feeder doors 40 times. In contrast, of 35 visitations recorded during the treatment period, raccoons opened feeder doors six times.

#### Carcass searches for target and non-target species

**Fig 5:**
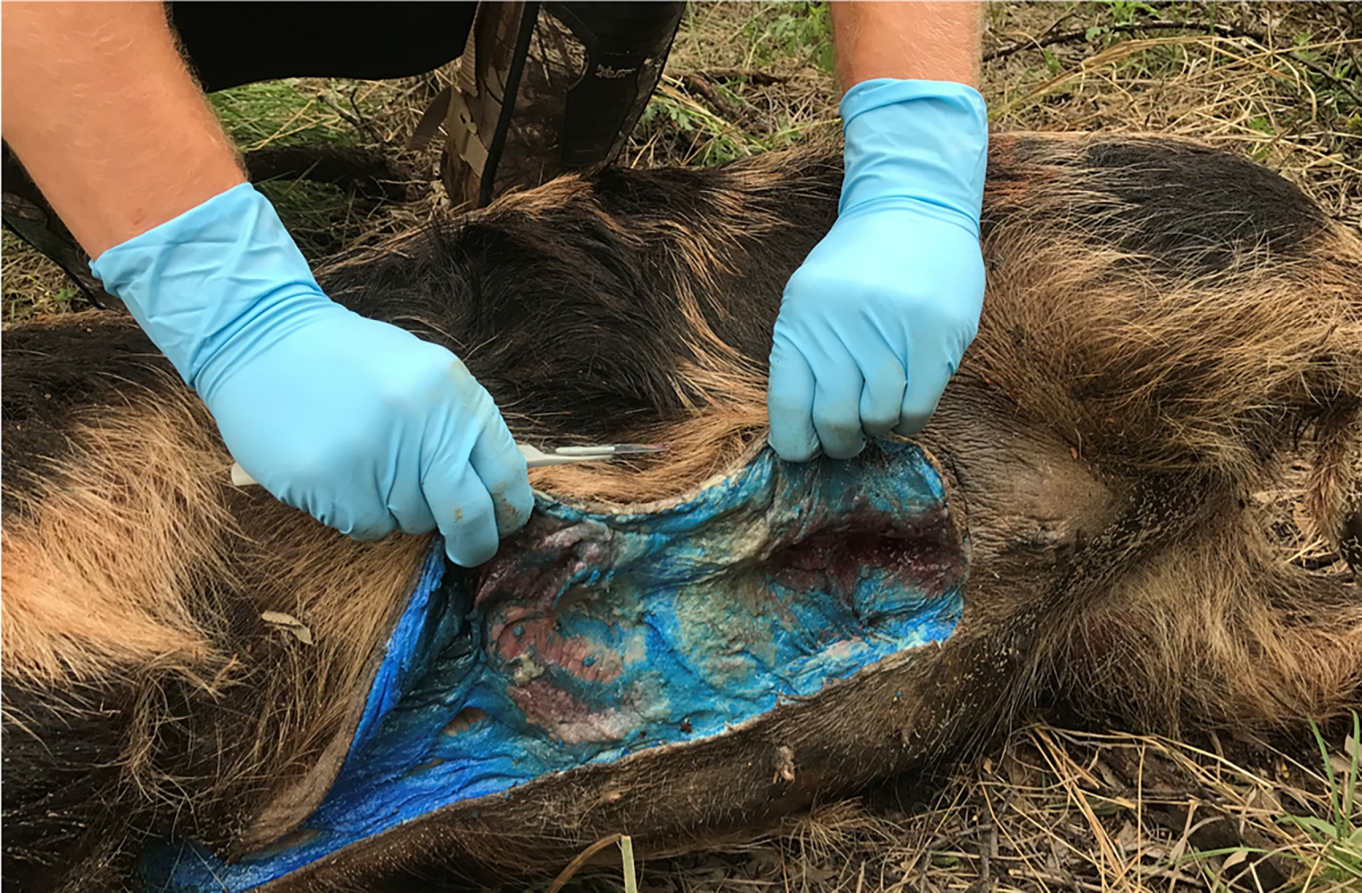
Wild pig succumbing to warfarin as evidenced by the blue subcutaneous fat, resulting from ingestion of the Keystone Blue dye.

Ninety-seven (97) carcass searches were conducted for wild pigs and non-target wildlife within the three plots during the treatment and post-treatment phases of the study. Thirty-five (35) wild pig carcasses were recovered as 18, 12, and 5 on the 0.005% and 0.01% warfarin-treated plots and control, respectively. Of the 18 wild pig carcasses recovered from the 0.005% warfarin plot, 17 showed signs of bait consumption evident by blue colored internal fat. Internal bleeding or bait in the gastrointestinal tract were signs of bait ingestion. Of the 12 wild pig carcasses recovered from the 0.01% warfarin plot, 11 showed signs of bait consumption. The two deceased pigs not showing signs of bait consumption within the 0.005% and 0.01% warfarin plots were killed by hunters, as evidenced by bullet wounds. Signs of bait consumption included internal bleeding and the presence of the blue dye (Fig 5). Of the five pigs recovered from the control plot, none showed signs of bait consumption. One was hit by a vehicle and four drowned during heavy flooding.

**Fig 6:**
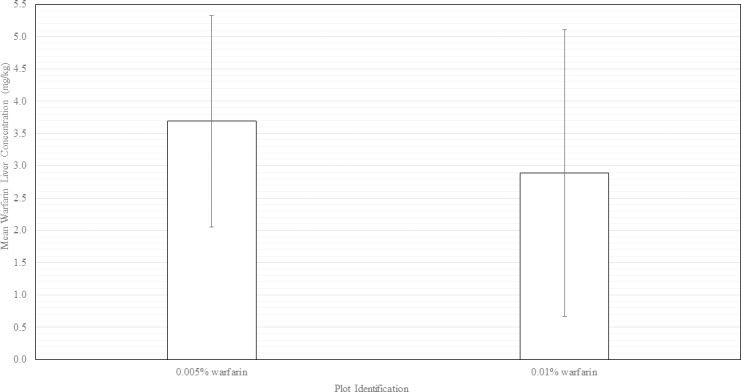
Mean warfarin residue concentrations (mg/kg) in liver samples detected via High Performance Liquid Chromatography (HPLC). Vertical bars indicate ± SE.

Twenty-three (23) wild pig liver samples were collected from carcasses recovered during the study. Of those obtained, 13 were from pigs recovered within the 0.005% warfarin plot and 9 from the 0.01% plot. All showed signs of bait consumption, blue dye, and had warfarin residues. These ranged from 0.42 mg/kg to 6.16 mg/kg, with a mean concentration of 3.69 ±1.7 mg/kg in the 0.005% warfarin plot (Fig 6). The warfarin residues from the 0.01% plot ranged from 0.93 mg/kg to 8.06 mg/kg, with a mean level of 2.89±2.35 mg/kg. No samples from pigs on control plot had warfarin residue nor signs of bait consumption.

#### Scavenging and decomposition

**Fig 7:**
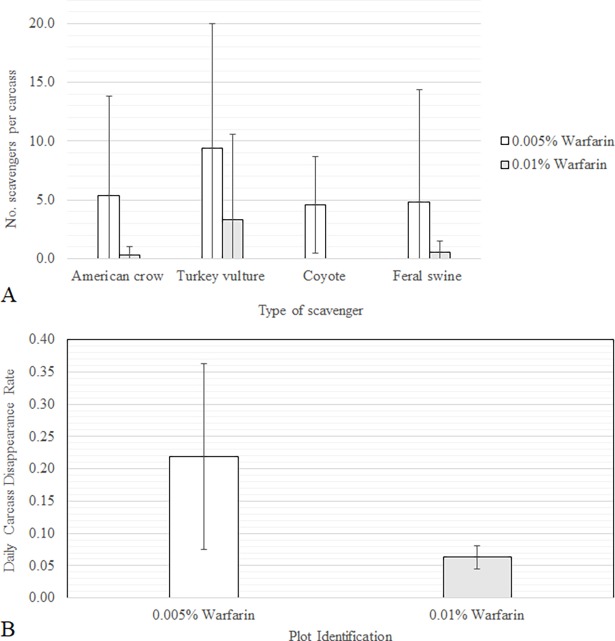
(A) The total number of non-targets observed on camera scavenging staged carcasses within each treatment plot; and (B) the mean days until disappearance of the carcasses within each treatment plot.

Scavenging was more prevalent within the 0.005% warfarin plot, resulting in quicker removal of the seven carcasses (2–8 days) relative to the five carcasses within the 0.01% warfarin plot (12–21 days) (Fig 7). Turkey vultures (*Cathartes aura*) were the main scavengers present at all carcass monitoring sites, comprising 48.5% and 92% of the scavengers present followed by American crows (*Corvus brachyrhynchos*) at 27.8% and 8% on the 0.005% and 0.01% warfarin plots respectively. Coyotes (*Canis latrans*) were observed only on the 0.005% plot in 23.7% images.

### Efficacy determinations

#### Radio telemetry

**Fig 8:**
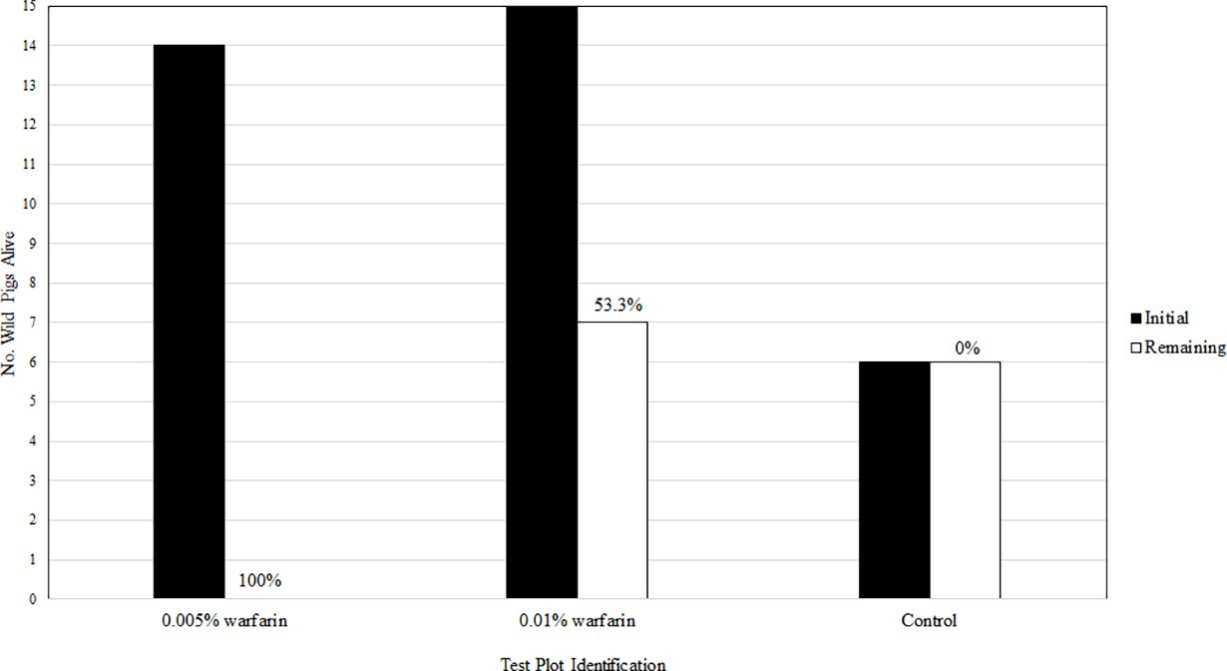
The efficacy of warfarin bait in reducing wild pig numbers within each plot.

All radio-tagged wild pigs on the 0.005% warfarin plot were found dead and showed signs of bait consumption, resulting in 100% efficacy (excluding those with detached ear tags or shot by hunters) (Fig 8). Of the 15 radio-tagged pigs on 0.01% warfarin treated plot included in the efficacy calculation, 8 were found dead showing signs of bait consumption and 7 remained alive, resulting in 53.3% efficacy. The efficacy of the 0.005% warfarin, in reducing radio-tagged wild pig abundance, exceeded the EPA requirements for product registration (70%) [54] and exceeded the estimated replacement rate (86%) [23].

#### Consumption

**Fig 9:**
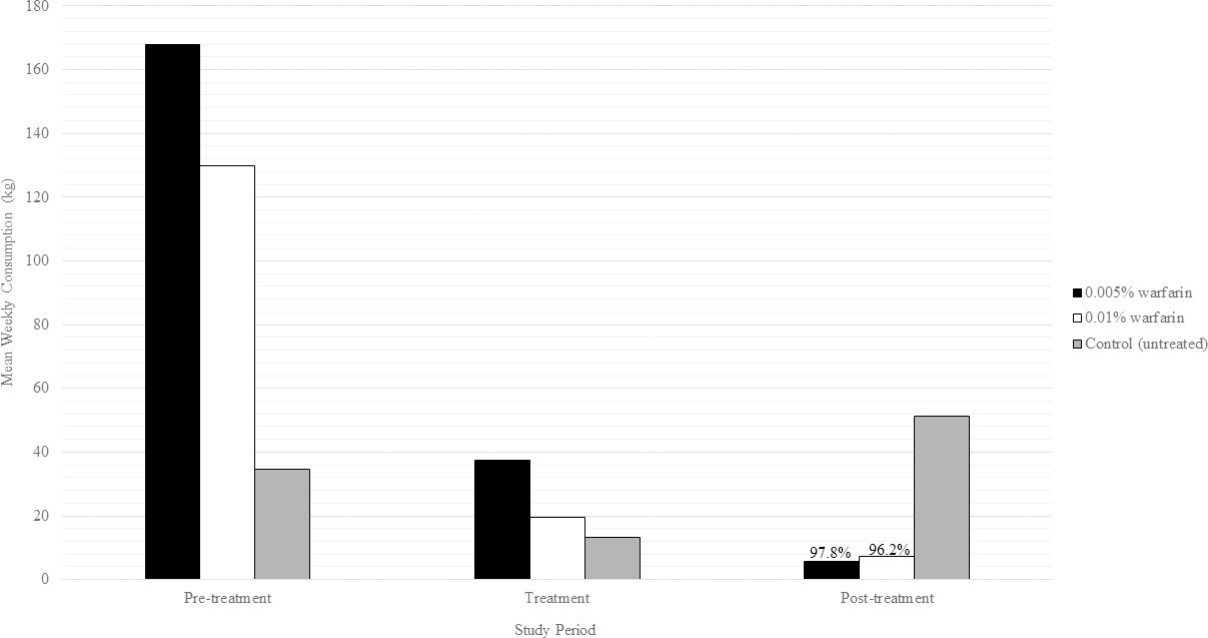
The average weekly consumption recorded during the pre-treatment, treatment and post-treatment periods, within the 0.005% and 0.01% warfarin-treated plots and the control plot.

Consumption of placebo within the control declined between pre-treatment (34.5 kg/week) and treatment (13.2 kg/week) but increased during post-treatment (51.4 kg/week) (Fig 9). Consumption within the 0.005% warfarin plot during pre-treatment, treatment and post-treatment decreased from 167.9 to 37.5 to 5.6 kg/week, respectively. The reduction from pre- to post-treatment was 97.8%. Consumption within 0.01% plot decreased during pre-treatment, treatment and post-treatment from 129.9 kg/week to 19.6 and 7.3 kg/week, respectively, with a reduction from pre- to post-treatment estimated to be 96.2%. The efficacy of the 0.005% and 0.01% warfarin baits, in reducing consumption by wild pigs, exceeded the EPA requirements for product registration (70%) [54] and exceeded the estimated replacement rate (86%) [29].

Weekly consumption within the control did not differ significantly when comparing pre-treatment, treatment, and post-treatment (ANOVA: *p* = 0.5903, *f*-*ratio* = 0.5376, *df* = 2), but was significantly different when comparing study periods within the 0.005% (ANOVA: *p* = 0.0004, *f*-*ratio* = 10.6602, *df* = 2) and 0.01% warfarin plots (ANOVA: *p* = 0.0015, *f*-*ratio* = 8.3295, *df* = 2), respectively. Significant differences between study periods were most evident when comparing pre-treatment to post-treatment within the 0.005% warfarin plot (Tukey’s: *p* = 0.0003) and the 0.01% warfarin plot (Tukey’s: *p* = 0.0016). Significant differences were also detected between pre-treatment and treatment consumption within baited plots (Tukey’s: *p* = 0.035, 0.015).

## Discussion

Wild pigs cause ~$2.5 billion in damage and control-related costs [55] and can transmit several zoonotic pathogens to man and livestock [56]. The results of this field study suggest that bait containing warfarin, at concentrations 80% less than commercial rodenticides (0.025%) [41,45], significantly reduce wild pig abundance within treated areas and could serve as a method of control. Both concentrations of warfarin (0.005% and 0.01%) proved effective in reducing pig numbers as demonstrated by radio-telemetry and bait consumption rates from data collected pre- and post-treatment. In this study, the 0.005% warfarin bait was more efficacious in reducing radio-tagged pig abundance (100% mortality). This may have resulted from a difference in terrain, which was rougher and rockier within the 0.01% treated plot. Feeder placement was more difficult since access to some areas was not feasible when transporting large equipment. We hypothesize that marked wild pigs surviving the 0.01% warfarin treatment period fled the area in response to the undesirable terrain and/or stress of being captured and marked prior to bait application. This study was performed on private land and was dependent on the permission of individual landowners, which limited our ability to account for movement of individual wild pigs following trapping and marking, and to relocate feeder clusters. Additionally, bait consumption and the mean warfarin residues in livers of killed pigs were greater within the 0.005% warfarin baited plot compared to the 0.01% plot. This suggests that individual pigs within the lower dose plot consumed far more bait and thus, the bait was more palatable than at the higher concentration. This would help further explain the greater efficacy in reducing radio tagged pigs within the 0.005% warfarin plot (100%) relative to the 0.01% treatment plot (53.3%). It is uncertain how much, if any, bait was consumed by surviving VHF tagged pigs, because they were unable to be euthanized post-treatment. These animals were wild and free-ranging and were unable to be collected immediately following the experiment. While euthanizing individuals post-study would be ideal, this would likely have to be performed under semi-controlled pen conditions to increase success.

It is worth noting that although camera counts proved useful in determining non-target feeder visitation during the pre-treatment and treatment periods, we did not use these data to determine efficacy in reducing wild pigs in this paper, instead focusing on radio-tagged individuals and feed consumption. This decision was made because the numbers within the control plot were noticeably low during the pre-treatment census (*n* = 1) and therefore we determined that an adequate baseline was not obtained. This likely resulted from the short duration of the pre-treatment census period (3-days). The EPA requires a >70% reduction in vertebrate pest activity, in at least two categories when applying for product registration [54]. Feed consumption and radio-telemetry are included as measurements of efficacy and therefore the results of the current study exceed the requirements outlined by the EPA. However, in future studies we recommend lengthening the pre-treatment census period to increase the probability of collecting more images of the target species and then using these images (pre-treatment vs. post-treatment) as another means of evaluating bait efficacy.

Data collected from pigs fitted with GPS technology can further assist managers in understanding what factors may influence landscape-use by wild pigs [57–59], which could aid in determining the placement of toxicant-baited feeders and the recovery of the carcasses of pigs succumbing to toxicants. The findings of [60] suggest that the average home range of wild pigs (15.13 ± 3.49 km^2^), at the study sites used in the current study, are comparable or smaller than some of the home range sizes in 1) other areas in Texas, 2) other wild pig-endemic U.S states, and 3) other pig-endemic countries, but that home range sizes in Davis Mountains, Texas (34 km^2^); the Northern Territory, Australia (24.1 km^2^); and in Kent Country, Texas (23.1 km^2^) might on average be larger than during the current study [61]. External factors such as nutrient availability [62], rainfall [63] and temperature [64] may influence wild pig home range size. However, a clear indication of how environmental variables influence home range sizes in different geographic foci has not been indicated [61]. It could be argued that warfarin bait may be advantageous in other areas where pig activity and home range sizes are known to be comparable or smaller than those in the current study area and the positioning of feeder clusters could be allocated based on this knowledge. However, given the global occurrence of wild pigs and the differences in average home range sizes, we recommend the replication of the above study under a variety of environmental conditions in differing geographic regions.

Raccoons were the only non-target wildlife species to open feeder doors, but none showed signs of bait consumption. However, to better exclude non-target species, useful modifications have recently been made to feeders, which include increasing the weight of the doors from ~4.5 to ~7.7 kg and utilizing guillotine-style doors [65]. Additionally, most non-target animals visiting feeders made no attempts to access the bait and relative interest in the feeders appeared to decline over the course of the study, as evidenced by the decrease in non-target visitations.

The application rate of warfarin during the treatment period was relatively low within two treatment plots at ~2.6 and ~4.5 g/km^2^, respectively, and spillage was minimal. The total warfarin spillage collected within the two treatment plots (~44 mg) was ~11x less than the estimated acute LD_50_ for a 10 kg dog (50 mg/kg) [43]. The cumulative treated area was ~16 km^2^, suggesting warfarin spillage amounted to ~2.75 mg/ km^2^ cumulatively over a 4-week period. Primary and secondary poisoning of wildlife pose little risk at low warfarin concentrations, and the dosages used within each treatment plot were 60 and 80% less than the dose found in EPA-registered, commercially available rodenticides [41].

Non-target wildlife would likely show no effects from consuming wild pig carcasses with warfarin residue. The highest individual concentration of warfarin residue observed (8.06 mg/kg) came from a sample collected within the 0.01% warfarin plot. At this warfarin residue, a 10 kg dog would need to eat >62 kg of pig liver to reach the estimated acute oral LD_50_ [43]. Chronic exposure is unlikely, as carcasses at decomposition monitoring stations were removed by scavenging activity within days. Vultures and crows were the most common scavengers observed, and the EPA has determined warfarin to be virtually non-toxic to birds and fish [41]. Laboratory studies in which European ferrets (*Mustela putorious furo*) and black-billed magpies (*Pica pica*) were fed black-tailed prairie dogs succumbing to warfarin bait at concentrations 10x the proposed wild pig concentration (500 ppm) demonstrated no adverse effects [66]. Recent findings suggest that insects alone can remove wild pig carcass tissues within several days, even during mass mortality events [67]. We should note that our study was conducted during the spring-summer months, and we were unable to determine rates of decomposition during the winter. During the winter, the rate of decomposition in swine would be expected to be reduced [68]. Our experimental use permit restricted us from applying the bait during the winter. However, future experiments should consider the rate of decomposition during this time for comparison with warmer periods. Regarding human safety, the importance of marking the pigs with blue dye should be reiterated, especially because consumption of anticoagulants by wild pigs has been identified as a possible means for anticoagulants to enter human foods [69]. The blue dye staining internal and subcutaneous fat served the purpose of confirming the consumption of the warfarin baits, thus reducing potential human risks by preventing the possible entry of warfarin into the human food chain. It is also encouraging to note that warfarin residues in recovered livers suggest reduced risk of warfarin to humans. Some patients taking warfarin medically take upwards to two 10 mg tablets per day (20 mg/day) [70]. At the highest liver warfarin concentration recorded in the current study, a human would need to eat >2 kg liver per day to reach this medically prescribed dose level.

The 0.005% warfarin bait evaluated during the current study was registered for use by the United States EPA (Kaput Feral Hog Bait, EPA Reg. No. 72500–26) in 2017 [71]. In the U.S., outdoor vertebrate pest control products are regulated under the EPA directive, the Federal Insecticide, Fungicide & Rodenticide Act (FIFRA) [72]. The EPA recommends using warfarin as a rodenticide for outdoor use at concentrations 80% greater than that of this wild pig bait [45] and does not consider the chemical inhumane. When initiating a vertebrate pest control program two important questions should be asked: 1) is the specific vertebrate control necessary, and 2) do the benefits outweigh the potential for harm [73]? First, given the global burden invasive wild pigs present, it’s difficult to argue against the need to control them, and the results of the current study suggest that 0.005% warfarin bait can reduce wild pig abundance markedly (>90%) in areas where it is applied. In response to concerns regarding potential non-target wildlife exposure, the concentration of warfarin deployed in the current study (0.005%) was ~26x (0.13%) and ~18x (0.09%) lower than that which was used in previous studies conducted in Australia. As mentioned previously, no non-target fatalities were observed after 4 weeks of bait exposure during the current study, nor were non-targets recovered in two years of subsequent field trials conducted in the same area in 2016 and 2017 [74]. By comparison, sodium nitrite (10%) formulations administered under field conditions have resulted in mortality of multiple non-target species including bird species, raccoons and a cow [75–76].

It should be noted that the number of sounders within each study plot were relatively unknown. Subsequently, explicit data representing the distribution and proportion of marked individuals within each sounder are not available. Given the natural social grouping of wild pigs [3,77], one can assume that all pigs within a sounder could be exposed to, and potentially eliminated by warfarin bait presented in feeders. This is very encouraging from a management standpoint, suggesting that an entire sounder might be removed by a single feeder cluster. However, it calls into question the reliability of the “individual pig” as an experimental unit. If a significant proportion of individuals within a sounder were marked, and hence used to estimate warfarin efficacy, this could create artificial inflation of the results. However, we can say with relative confidence that wild pigs from multiple sounders were collared within each plot, based on observations made via motion-sensitive trail cameras, and that most pigs within individual sounders remained unmarked. Additionally, it is beneficial that multiple methods were used to evaluate efficacy and was encouraging that feed consumption was reduced by >90% within both the 0.01% and 0.005% warfarin baited plots, exceeding EPA recommendations [54]. While we hypothesize that the efficacy of the 0.005% warfarin bait in reducing marked pigs and feed consumption are reflective of the larger population within the plot, we recognize the above caveat and suggest appropriate modifications be made during future studies. Study limitations are not surprising, given this is the first wild pig toxicant field study to be conducted in North America. During future warfarin bait field trials, researchers should strive to estimate the number and size of sounders prior to study initiation, possibly using methods similar to those described by [78], and an effort should be made to distribute GPS collars and VHF ear tags amongst them with proportional homogeneity. This could potentially improve efficacy estimates and the capacity to analyze results statistically.

## Conclusions

This study is the first field evaluation of a toxicant for use against wild pigs in the U.S. and suggests that bait containing 0.005% warfarin may be effective in reducing wild pig numbers within treated areas and suggests that the study be replicated to further determine the impacts on wild pig populations in Texas. During future studies, researchers should consider employing methods to better estimate the number of pig sounders within study areas. Given the global issue that wild pigs represent, we also recommend further trials be conducted in other regions to replicate the results under different climatic and geographic conditions. The relatively low bait application rate, warfarin liver concentrations, and spillage indicate that this product could be an environmentally conscious control measure. The Keystone Blue dye proved efficacious in marking wild pigs consuming bait, which could serve as a valuable tool for alerting hunters of warfarin-bait consumption or of other toxicants which might be formulated with the dye. Studies are currently underway which confirm efficacy of the warfarin bait, show improvement of the feeders by ensuring access is confined to pigs only, and supplement wild pig movements via GPS tracking. The fact that wild pigs are a globally expanding issue suggests that low-dose warfarin baits, may provide another management tool to effectively control this invasive species in other areas in Texas, the southeastern United States, and beyond.

## Supporting information

S1 Table (A) Total wild pigs fitted with transmitters, (B) Consumption per study period, per plot, per cluster, (C) Non-target wildlife visitations, (D) Feeder access by raccoons, (E) Warfarin residues in livers of deceased wild pigs.
(XLSX)
